# Supplementary material for: Partial FAM19A5 deficiency in mice leads to disrupted spine maturation, hyperactivity, and an altered fear response
Source: PLoS One. 2025 Aug 5;20(8):e0327493. doi: 10.1371/journal.pone.0327493 (PMC12324117; doi:10.1371/journal.pone.0327493)
Supplement: S1 Fig — (A) Western blot analysis of the time-dependent degradation of the wild-type (WT) and mutant (MT) FAM19A5 proteins, and beta-actin. Changes in protein levels were observed at various time points after co-treating cells with either cycloheximide (CHX) alone or CHX and MG132 simultaneously. (B, C) Quantification of the (B) FAM19A5 protein bands (n = 3 each, comparison of linear regression models, slope: F(3, 40) = 0.1966, P = 0.8981), and (C) beta-actin (n = 6 each, Two way ANOVA followed by Šídák’s multiple comparisons test, **p = 0.0066). (DOCX) [file pone.0327493.s001.docx]

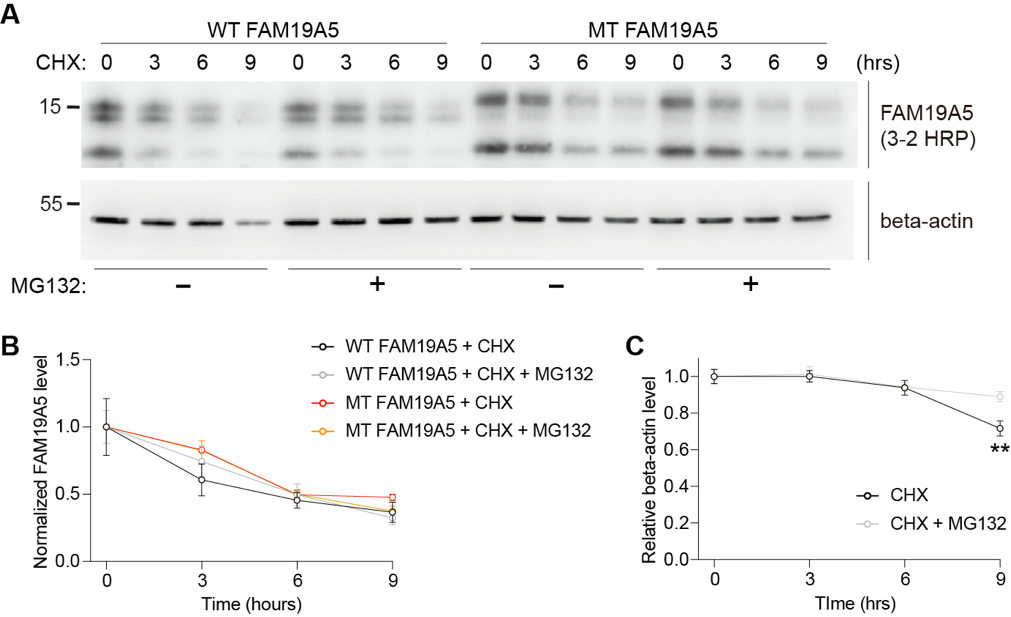


**Supplementary Fig 1. Time-dependent degradation of FAM19A5 protein in HEK293 cells.**

(A) Western blot analysis of time-dependent degradation of wild-type (WT) and mutant (MT) FAM19A5 proteins, and beta-actin. Changes in protein levels were observed at various time points after co-treating cells with either cycloheximide (CHX) alone or CHX and MG132 simultaneously. (B, C) Quantification of the (B) FAM19A5 protein bands (n = 3 each, comparison of linear regression models, slope: F(3, 40) = 0.1966, P=0.8981), and (C) beta-actin (n=6 each, Two way ANOVA followed by Šídák's multiple comparisons test, **p=0.0066).
