# Supplementary material for: Partial FAM19A5 deficiency in mice leads to disrupted spine maturation, hyperactivity, and an altered fear response
Source: PLoS One. 2025 Aug 5;20(8):e0327493. doi: 10.1371/journal.pone.0327493 (PMC12324117; doi:10.1371/journal.pone.0327493)
Supplement: S2 Fig — (A) Nissl-stained brain slices.The motor, somatosensory, visual, and auditory cortical regions are indicated by the black box. Scale bar, 1 mm. (B) Layers 1–6 of the motor, somatosensory, visual, and auditory cortex in WT, heterozygous, and homozygous LacZ KI mice. Scale bar, 50 μm. (C) Quantification of cortical layer thickness (n = 5). Data are presented as mean ± SEM. (DOCX) [file pone.0327493.s002.docx]

**
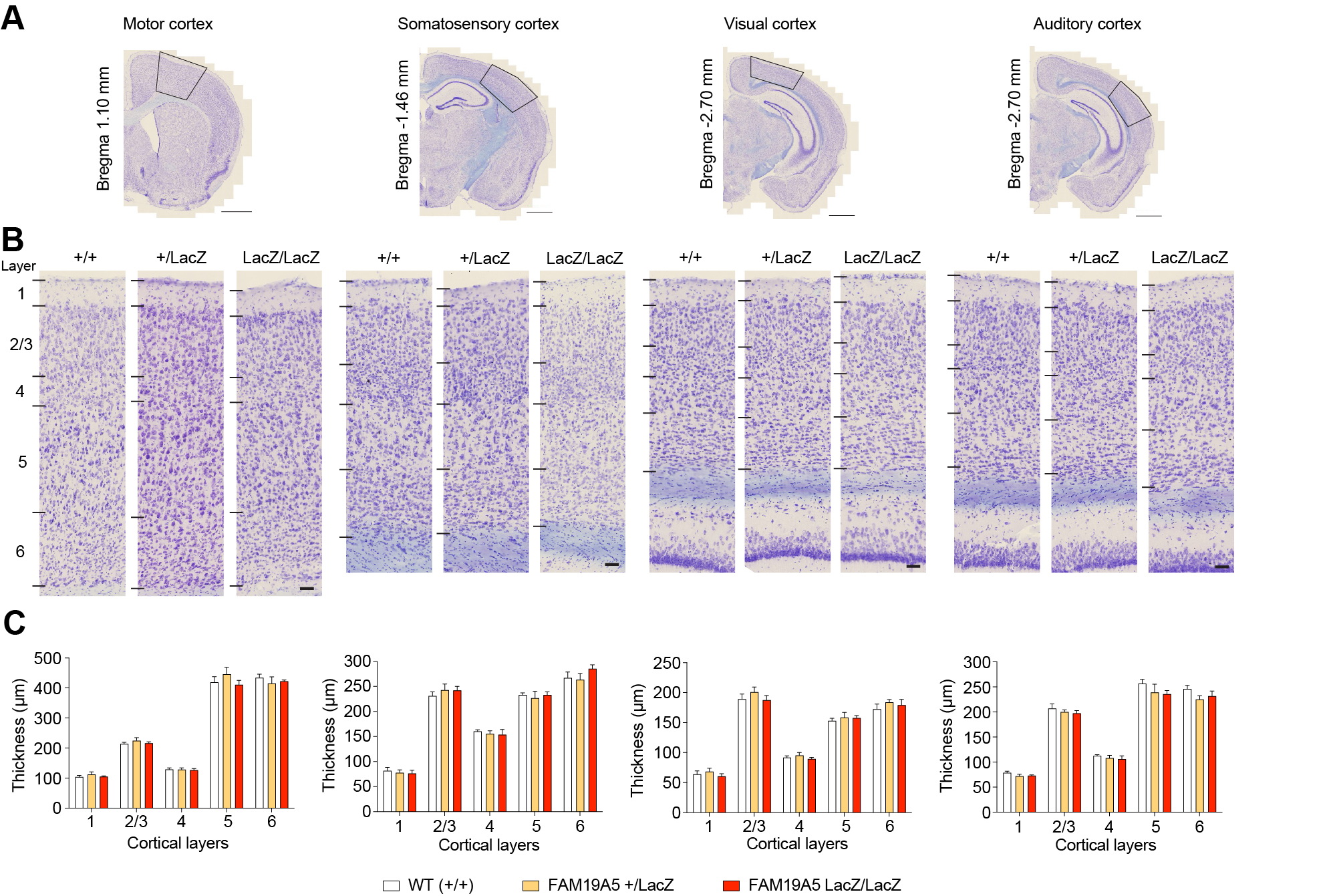
**

**Supplementary Fig 2. Characterization of cortical layers in FAM19A5 LacZ KI mice.**

(A) Nissl-stained brain slices. Motor, somatosensory, visual, and auditory cortical regions are indicated by the black box. Scale bar, 1 mm. (B) Layers 1-6 of the motor, somatosensory, visual, and auditory cortex in WT, heterozygous, and homozygous LacZ KI mice. Scale bar, 50 μm. (C) Quantification of cortical layer thickness (n=5). Data are presented as mean ± SEM.
