# Supplementary material for: Partial FAM19A5 deficiency in mice leads to disrupted spine maturation, hyperactivity, and an altered fear response
Source: PLoS One. 2025 Aug 5;20(8):e0327493. doi: 10.1371/journal.pone.0327493 (PMC12324117; doi:10.1371/journal.pone.0327493)
Supplement: S3 Fig — (A) Total number of rearings, (B) supported rearing, (C) unsupported rearing, and (D) total grooming time during the 10 min of exploration period in the OFT. FAM19A5+/+, n = 13 and FAM19A5LacZ/LacZ, n = 15. Data are presented as the mean ± SEM. **P < 0.01 vs. FAM19A5+/+. (E) Latency to fall in a rotating rod during 3 trials per day for 5 consecutive days (FAM19A5+/+, n = 9 and FAM19A5LacZ/LacZ, n = 7). (F) Latency to fall in a hanging wire test in 300 s long 3 consecutive trials with at least 15 min intertrial interval (FAM19A5+/+, n = 18 and FAM19A5LacZ/LacZ, n = 16). (G) Number of marbles buried during the 30 min exploration time (FAM19A5+/+, n = 14; FAM19A5+/LacZ, n = 9; FAM19A5LacZ/LacZ, n = 14). (H) Percentage of shredded nestlet during 30 min of observation time (FAM19A5+/+, n = 16; FAM19A5+/LacZ, n = 13; FAM19A5LacZ/LacZ, n = 15). Data are presented as the mean ± SEM. ***P < 0.001 vs. FAM19A5+/+. (DOCX) [file pone.0327493.s003.docx]

**
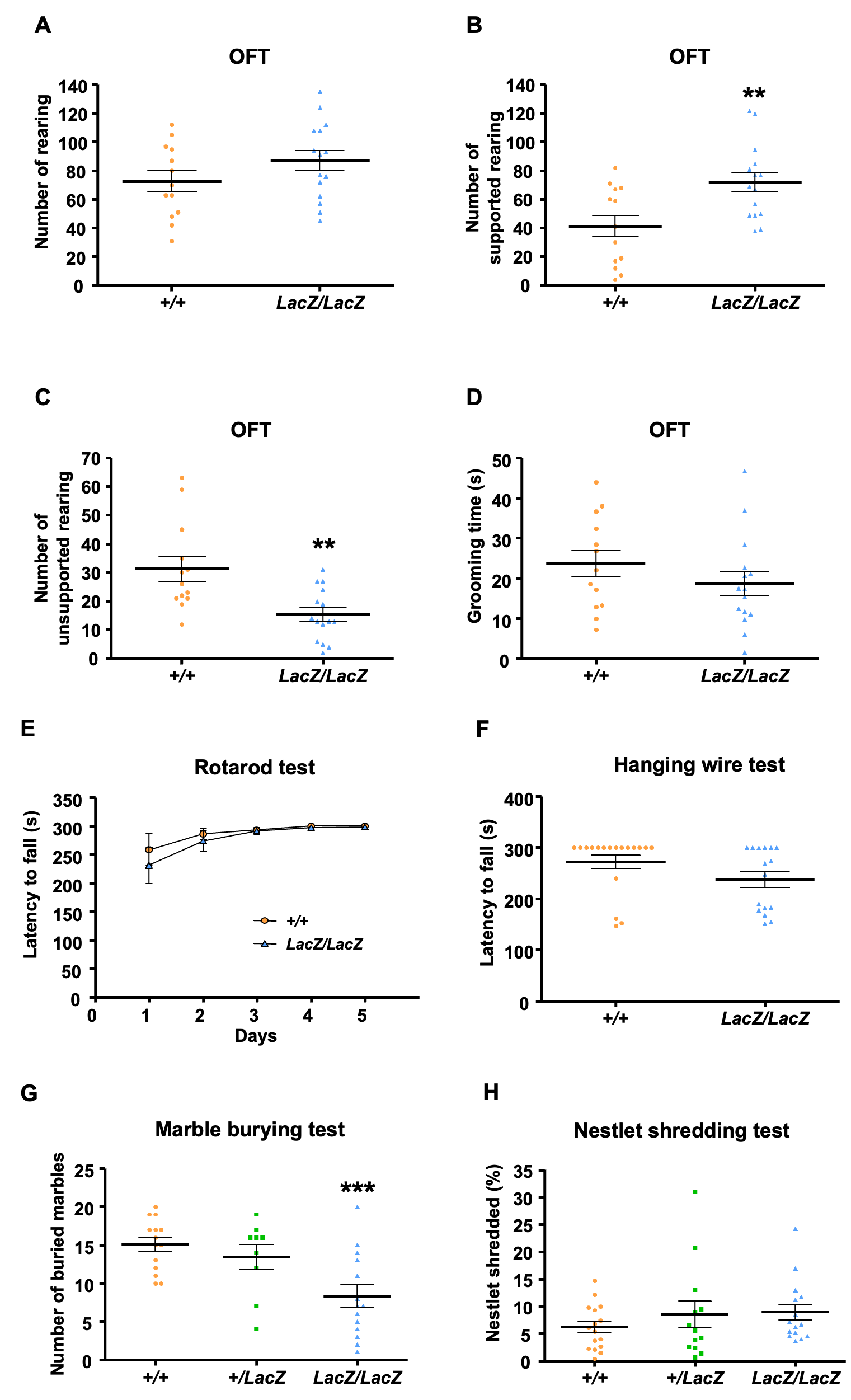
**

**Supplementary Fig 3.** **Rearing and grooming behavior in FAM19A5-LacZ KI mice.**

(A) Total number of rearing, (B) supported rearing, (C) unsupported rearing, and (D) total grooming time during 10 min of exploration period in OFT. FAM19A5^+/+,^ n=13 and FAM19A5^LacZ/LacZ^, n=15. Data are presented as the mean ± SEM. **P<0.01 vs. FAM19A5+/+. **(**E) Latency to fall in a rotating rod during 3 trials per day for 5 consecutive days (FAM19A5^+/+^, n=9 and FAM19A5^LacZ/LacZ^, n=7). (F) Latency to fall in a hanging wire test in 300 s long 3 consecutive trials with at least 15 min intertrial interval (FAM19A5^+/+^, n=18 and FAM19A5^LacZ/LacZ^, n=16). (G) Number of marble buried during 30 min of exploration time (FAM19A5^+/+^, n=14; FAM19A5^+/LacZ^, n=9; FAM19A5^LacZ/LacZ^, n=14). (H) Percentage of shredded nestlet during 30 min of observation time (FAM19A5^+/+^, n=16; FAM19A5^+/LacZ^, n=13; FAM19A5^LacZ/LacZ^, n=15). Data are presented as the mean ± SEM. ***P<0.001 vs. FAM19A5^+/+^.
