# Supplementary material for: Partial FAM19A5 deficiency in mice leads to disrupted spine maturation, hyperactivity, and an altered fear response
Source: PLoS One. 2025 Aug 5;20(8):e0327493. doi: 10.1371/journal.pone.0327493 (PMC12324117; doi:10.1371/journal.pone.0327493)
Supplement: S4 Fig — (A) Representative track plot of movements in the elevated plus maze during 15 min of exploration time in FAM19A5LacZ/LacZ and FAM19A5+/+ littermates. (B and C) Total distance traveled and mean speed of movement in the elevated plus maze, respectively. (D and E) Percentage of time spent and total number of entries into the open arms of the maze, respectively. (F) Percentage of immobility time during the 5 min long TST. FAM19A5+/+, n = 16; FAM19A5+/LacZ, n = 12 and FAM19A5LacZ/LacZ, n = 16. Data are presented as the mean ± SEM. *P < 0.05 and **P < 0.01 vs. FAM19A5+/+. (DOCX) [file pone.0327493.s004.docx]

**
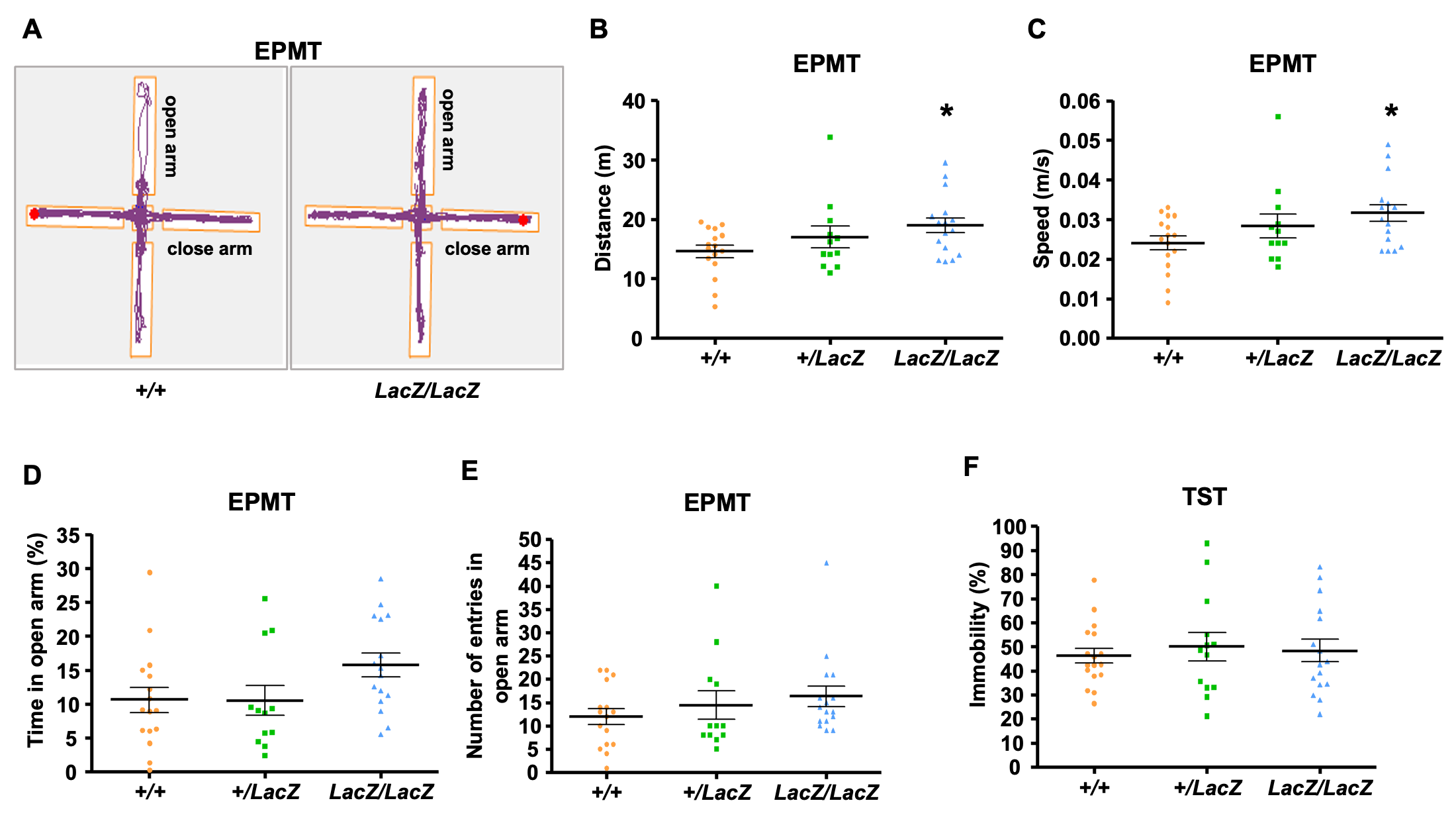
**

**Supplementary Fig 4.** **The absence of anxiety and depressive-like behavior in FAM19A5-LacZ KI mice.**

(A) Representative track plot of movements in elevated plus maze during 15 min of exploration time in FAM19A5^LacZ/LacZ^ and FAM19A5^+/+^ littermates. (B and C) Total distance traveled and mean speed of movement in elevated plus maze, respectively. (D and E) Percentage of time spent and total number of entries in open arm of the maze, respectively. (F) Percentage of immobile time during 5 min long TST. FAM19A5^+/+^, n=16; FAM19A5^+/LacZ^, n=12 and FAM19A5^LacZ/LacZ^, n=16. Data are presented as the mean ± SEM. *P<0.05 and **P<0.01 vs. FAM19A5^+/+^.
