# Supplementary material for: Partial FAM19A5 deficiency in mice leads to disrupted spine maturation, hyperactivity, and an altered fear response
Source: PLoS One. 2025 Aug 5;20(8):e0327493. doi: 10.1371/journal.pone.0327493 (PMC12324117; doi:10.1371/journal.pone.0327493)
Supplement: S5 Fig — (A-C) Total distance traveled, speed of motion and percentage of spontaneous alteration in Y-maze arms during 5 min of exploration period in FAM19A5+/LacZ, n = 12; FAM19A5LacZ/LacZ, n = 16 and FAM19A5+/+, n = 16. (D) Percentage of preference to novel object during 10 min exploratory time in NOR test after 6 h for short term memory and (E) after 24 h for long term memory. FAM19A5+/+, n = 7; FAM19A5+/LacZ, n = 3; FAM19A5LacZ/LacZ, n = 8 for short term memory and FAM19A5+/+, n = 5; FAM19A5+/LacZ, n = 4; FAM19A5LacZ/LacZ, n = 7 for long term memory. Data are presented as the mean ± SEM. *P < 0.05 vs. FAM19A5+/+. (DOCX) [file pone.0327493.s005.docx]

**
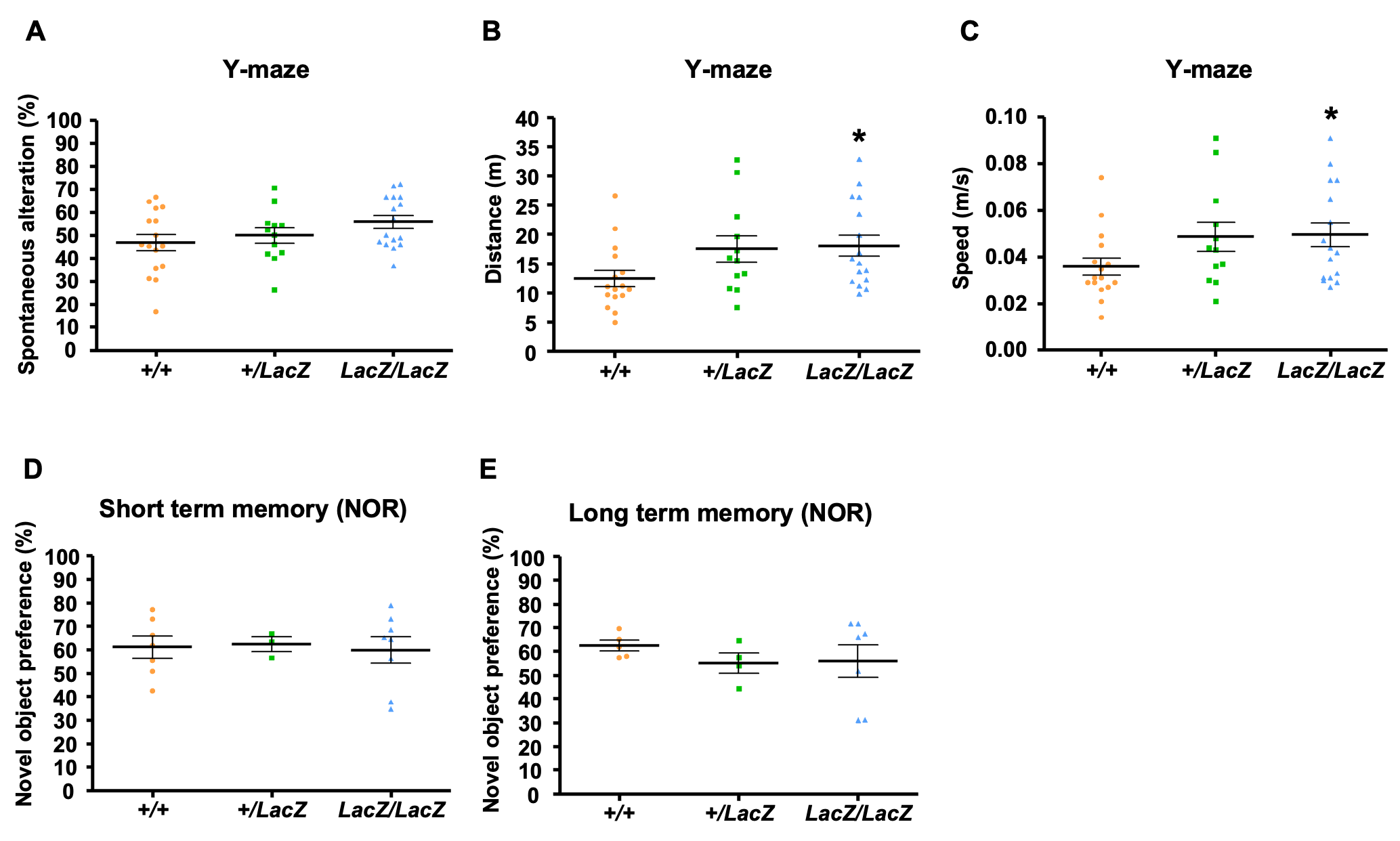
**

**Supplementary Fig 5.** **Normal learning and memory function in FAM19A5-LacZ KI mice.**

(A-C) Total distance traveled, speed of motion and percentage of spontaneous alteration in Y-maze arms during 5 min of exploration period in FAM19A5^+/LacZ^, n=12; FAM19A5^LacZ/LacZ^, n=16 and FAM19A5^+/+,^ n=16. (D) Percentage of preference to novel object during 10 min exploratory time in NOR test after 6 h for short term memory and (E) after 24 h for long term memory. FAM19A5^+/+^, n=7; FAM19A5^+/LacZ^, n=3; FAM19A5^LacZ/LacZ^, n=8 for short term memory and FAM19A5^+/+^, n=5; FAM19A5^+/LacZ^, n=4; FAM19A5^LacZ/LacZ^, n=7 for long term memory. Data are presented as the mean ± SEM. *P<0.05 vs. FAM19A5^+/+^.
