# Supplementary figures and images for: Partial FAM19A5 deficiency in mice leads to disrupted spine maturation, hyperactivity, and an altered fear response
Source: PLoS One. 2025 Aug 5;20(8):e0327493. doi: 10.1371/journal.pone.0327493 (PMC12324117; doi:10.1371/journal.pone.0327493)

**Fig 1C**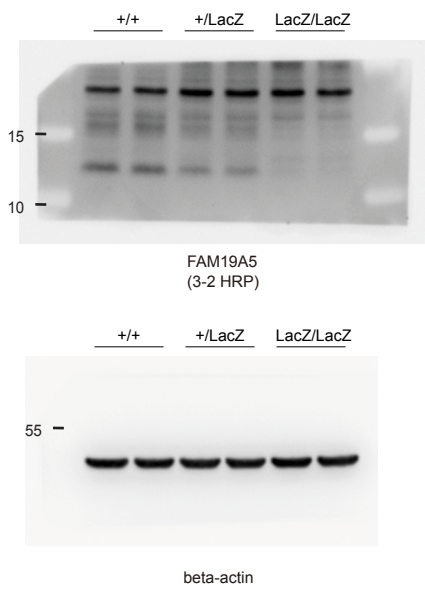**Fig 1F**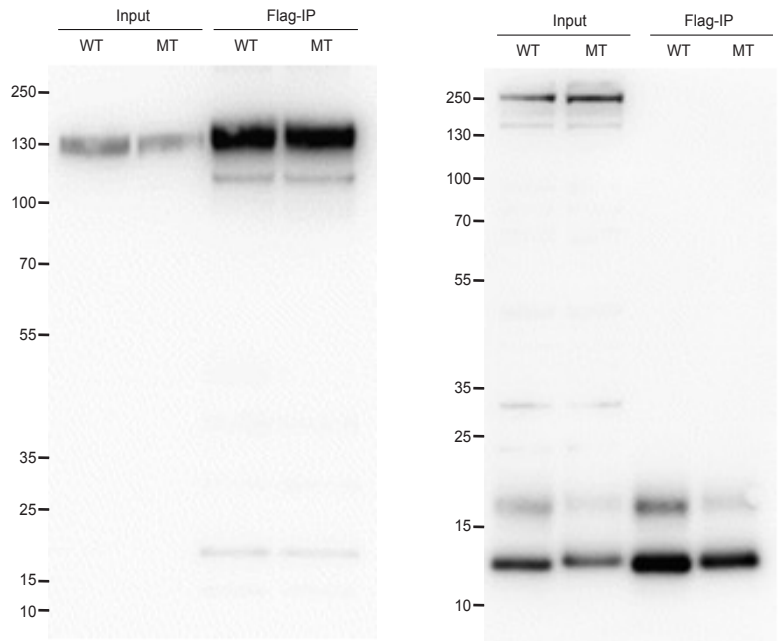**Fig S1A**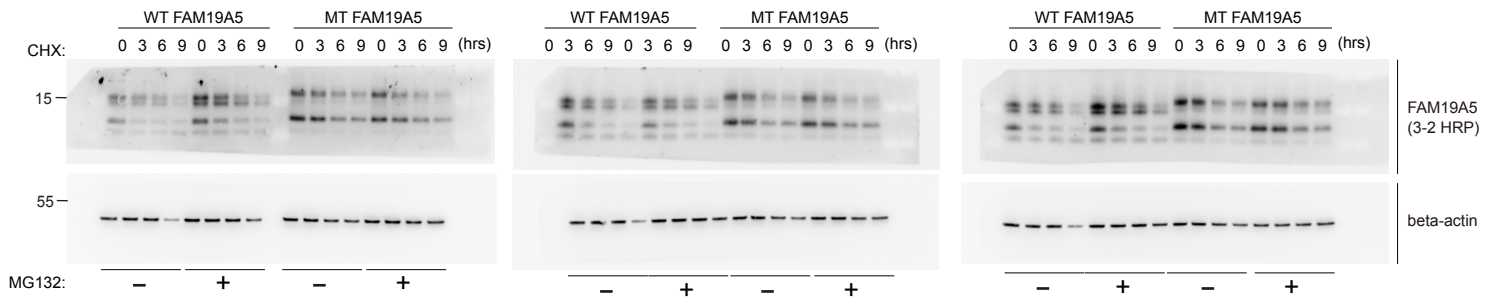

Supplement: S6 Raw gel images — (PDF) [file pone.0327493.s006.pdf]
