# Supplementary material for: Partial FAM19A5 deficiency in mice leads to disrupted spine maturation, hyperactivity, and an altered fear response
Source: PLoS One. 2025 Aug 5;20(8):e0327493. doi: 10.1371/journal.pone.0327493 (PMC12324117; doi:10.1371/journal.pone.0327493)
Supplement: Attachment — Submitted filename: Respone to reviwer.docx [file pone.0327493.s009.docx]

**Response to Editor:**

**Figure1SA westernblot showed a clear splice mark between WT48h and MT0h. Apparently, the results were from two gel runs. If so, they should not be put together into one panel. Separate gel runs should be presented by different panels.**

Thank you for pointing that out. As the reviewer suggested, we have re-performed the experiment investigating the FAM19A5 protein degradation mechanism, which corresponds to Figure S1. The time-course experiment was conducted on a single gel; however, replicate experiments were performed on separate, independent gels. Please refer to the revised Figure S1.

**Figure 1SB, D, the term "the relative protein expression" should be explained in the method section.**

Thank you for your valuable feedback. The quantitative data obtained from three independent experiments is presented in Figure S1 B and C. To address the varying initial FAM19A5 levels between the experiments, we have normalized the FAM19A5 quantifies at their respective 0-hour time points. This ensures a more accurate comparison of degradation rates. Furthermore, we have updated the experimental protocol and added a detailed description of quantification methods for Figure S1B to the Methods section. Please refer to lines 112-121 in the revised manuscript.

**Response to Reviewer:**

**The authors have addressed all my previous comments. However, I have a follow-up regarding Fig S1, which was generated in response to my previous comment regarding the low levels of MT FAM19A5 in the lacZ KI mice. The authors used MG132 to explore this pathway, and the results are shown in Figs1.**

**The authors transfected the MT FAM19A5 protein in HEK 293, yet the protein migrates at the same molecular weight as the WT. Similarly, in Fig 1F, both WT and MT appear to run at the same level. However, in the updated version of Fig 1C, the authors stated that the WB analysis reveals a minor size difference in non-glycosylated MT FAM19A5 due to the additional 8 a.a at the C-term. It remains unclear whether the band represented by the open arrow in Fig1C represents non-glycosylated MT FAM19A5 from FAM19A5 LacZ/LacZ mice.**

We appreciate reviewer’s comments, but we partially agree with them. In our Western blot analysis (Fig 1C), a slightly higher migrating band observed exclusively in FAM19A5 LacZ/LacZ mice, compared to wild-type mice, is presumed to represent non-glycosylated MT FAM19A5. However, to avoid making a definitive claim, we intentionally used the term *“potential”* to moderate our interpretation. This revision is reflected in the updated text (lines 260-262) and Figure 1 legend.

We respectfully disagree with the reviewer’s statement that MT FAM19A5 in HEK293 cells migrates at the same size as WT FAM19A5. While the size difference in Figure 1F is subtle and not fully distinguishable, we believe a discernible shift is present. This observation has been consistently replicated in additional Western blot experiments, which further support a slight difference in size between the two forms. As shown in the image provided below, extending the gel running time results in a clearly distinguishable separation between non-glycosylated MT FAM19A5 and the WT protein.

Non-glycosylated MT FAM19A5 and WT FAM19A5 are more distinctly separated with extended gel electrophoresis. Solid arrowhead for glycosylated FAM19A5, Open arrowhead for non-glycosylated FAM19A5.

**Additionally, in Fig 1s, it is unclear why, at the 0h-timepoint, the CHX-treated group shows high levels of both WT and MT, while in the MG132-treated group, WT protein levels appear low and MT starts at higher levels. The increased accumulation of WT protein over time may be an artifact due to the initially low signal at 0h.**

**Clarification is also needed on how the blots were quantified as the quantification in panel D doesn’t seem to reflect the WB shown in panel C. How many independent experiments were performed to generate these results?
I suggest combining the two experiments into a single protocol in which cells are first treated with CHX to prevent additional protein synthesis and then with MG132 for 48 hours. This would allow for a more accurate assessment of protein half-life rather than relying solely on relative protein expression. If MG132 stabilizes the MT FAM19A5, it would support the author's hypothesis regarding protein degradation pathways. However, However, this is only a recommendation, the authors may choose to either conduct this revised experiment or clarify the current text accordingly.**

Thank you for your comments. As you recommended, we investigated the degradation kinetics of WT and MT FAM19A5 proteins by treating HEK293 cells overexpressing either WT or MT FAM19A5 with cycloheximide (CHX) to inhibit protein synthesis. Additionally, we included a group co-treated with MG132 to examine the involvement of the ubiquitin-proteasome pathway, a major route for protein degradation.

Upon CHX treatment at 0 hr, both FAM19A5 proteins degraded linearly over time. The half-lives of WT and MT FAM19A5 were similar, approximately 10.13 and 9.13 hours, respectively. When CHX and MG132 were co-administered, the degradation patterns of both proteins showed similar trends to those observed with CHX treatment alone, with half-lives of 10.94 and 9.45 hours, respectively, showing no significant difference. Conversely, beta-actin, which degraded slowly with CHX alone, remained stable and showed minimal degradation upon co-administration with MG132.

We replicated this experiment three times, and all yielded consistent results. These findings suggest that the degradation of both WT and MT FAM19A5 may proceed via alternative pathways rather than the conventional ubiquitin-proteasome pathway. However, we acknowledge that this *in vitro* experiment has limitations in fully reflecting the *in vivo* processes in the brain, thus necessitating further investigation.

We have incorporated these results into Figure S1 and updated the corresponding description in the main text, which can be found in lines 266-272. Furthermore, we have updated the experimental methods in the Materials and methods section and included the quantification approach you requested, which can be found in lines 112-121.

**Supplementary Figure 1. Time-dependent degradation of FAM19A5 protein in HEK293 cells.** (A) Western blot analysis of time-dependent degradation of wild-type (WT) and mutant (MT) FAM19A5 proteins, and beta-actin. Changes in protein levels were observed at various time points after co-treating cells with either cycloheximide (CHX) alone or CHX and MG132 simultaneously. (B, C) Quantification of the (B) FAM19A5 protein bands (n = 3 each, comparison of linear regression models, slope: F(3, 40) = 0.1966, P=0.8981), and (C) beta-actin (n=6 each, Two way ANOVA followed by Šídák's multiple comparisons test, **p=0.0066).

**Lastly, in Fig 1S A, the WT blot is cropped at the 48h time point, please ensure that the full band is visible.**

As explained above, we have replaced Fig. S1 with a revised version that was conducted using a single gel. Please refer to the updated Fig. S1.

**Line 272, remove the letter s from "(Figs 1C)"**

We've removed the letter "s" as reviewer suggested. Please see the change on line 262 of the revised manuscript.
